# Anti-tumor and anti-angiogenic effects of Fucoidan on prostate cancer: possible JAK-STAT3 pathway

**DOI:** 10.1186/s12906-017-1885-y

**Published:** 2017-08-01

**Authors:** Xin Rui, Hua-Feng Pan, Si-Liang Shao, Xiao-Ming Xu

**Affiliations:** Department of Urology, Ningbo No.2 Hospital, 41 Xibei Street, Ningbo, Zhejiang Province, 315010 China

**Keywords:** Fucoidan, Prostate cancer, Angiogenesis, STAT3

## Abstract

**Background:**

Prostate cancer is the most common cancer in men in the United States. Fucoidan is a bioactive polysaccharide extracted mainly from algae. The aim of this study was to investigate anti-tumor and anti-angiogenic effects of fucoidan in both cell-based assays and mouse xenograft model, as well as to clarify possible role of JAK-STAT3 pathway in the protection.

**Methods:**

DU-145 human prostate cancer cells were treated with 100–1000 μg/mL of fucoidan. Cell viability, proliferation, migration and tube formation were studied using MTT, EdU, Transwell and Matrigel assays, respectively. Athymic nude mice were subcutaneously injected with DU-145 cells to induce xenograft model, and treated by oral gavage with 20 mg/kg of fucoidan for 28 days. Tumor volume and weight were recorded. Vascular density in tumor tissue was determined by hemoglobin assay and endothelium biomarker analysis. Protein expression and phosphorylation of JAK and STAT3 were determined by Western blot. Activation of gene promoters was investigated by chromatin Immunoprecipitation.

**Results:**

Fucoidan could dose-dependently inhibit cell viability and proliferation of DU-145 cells. Besides, fucoidan also inhibited cell migration in Transwell and tube formation in Matrigel. In animal study, 28-day treatment of fucoidan significantly hindered the tumor growth and inhibited angiogenesis, with decreased hemoglobin content and reduced mRNA expression of CD31 and CD105 in tumor tissue. Furthermore, phosphorylated JAK and STAT3 in tumor tissue were both reduced after fucoidan treatment, and promoter activation of STAT3-regulated genes, such as *VEGF*, *Bcl-xL* and *Cyclin D1*, was also significantly reduced after treatment.

**Conclusions:**

All these findings provided novel complementary and alternative strategies to treat prostate cancer.

## Background

Prostate cancer is the most common cancer in men and the second leading cause of death from cancer in men in the United States [1, 2]. Many risk factors, such as genetic, dietary, medication exposure, infectious disease and sexual factors, can lead to the development of prostate cancer [3]. The therapy for prostate cancer usually involves a combination of surgery, chemotherapy and radiotherapy, however, the adverse effect is obvious [4, 5]. On the contrary, bioactive ingredients extracted from food resources can provide complementary and alternative strategies to treat prostate cancer [6].

Angiogenesis is the physiological or pathological process through which new blood vessels form from pre-existing vessels [7]. Angiogenesis does not initiate malignancy but promotes tumor progression and metastasis, therefore, intensive efforts have been undertaken to develop therapeutic strategies to inhibit angiogenesis in cancer over the past decades [7]. Signal transducers and activators of transcription 3 (STAT3) is a member of the STAT protein family. In response to cytokines and growth factors, STAT3 is phosphorylated by receptor-associated Janus kinases (JAK), then form homo or heterodimers, and translocate to the cell nucleus where they act as transcription activators [8, 9]. The abnormal activation of STAT3 can cause unrestricted cell proliferation, malignant transformation and tumor angiogenesis [8, 10]. Activation of STAT3 signaling is essential in the metastatic progression of prostate cancer, and targeting STAT3 pathway can yield a potential therapeutic intervention for prostate cancer [11–13].

Fucoidan is a sulfated polysaccharide obtained mainly in various species of brown algae and brown seaweed such as *Undaria pinnatifida*, *Laminaria angustata*, *Fucus vesiculosus*, and *Fucus evanescens* [14, 15]. It is reported that fucoidan has anti-tumor activity on lung, breast, liver, colon, prostate and bladder cancer cells [16]. Compared to medications, fucoidan is food-grade ingredient which can provide complementary and alternative strategies without intolerable side effects [17, 18]. In previous study, fucoidan induced the apoptosis of PC-3 human prostate cancer cells in vitro, but the possible role in vivo was still unknown [19]. Therefore, here we investigated anti-tumor and anti-angiogenic effects of fucoidan in both cell-based assays and mouse xenograft model, as well as tried to clarify a role of JAK-STAT3 pathway in the protection.

## Methods

### Reagents

Fucoidan was purchased from Sigma-Aldrich (St. Louis, MO). Fucoidan powder was dissolved in phosphate buffer saline (PBS), then sterilized using a 0.22 μm pore filter (Millipore, Billerica, MA) and stored at 4 °C until use.

### Cell culture

DU-145, androgen-independent human prostate carcinoma cells, were purchased from American Type Culture Collection (ATCC, Manassas, VA), and were grown in Modified Eagle’s Medium (MEM) supplemented with 10% fetal bovine serum and 1% penicillin/streptomycin (Gibco, Grand Island, NY) at 37 °C in a humidified 5% CO_2_ atmosphere.

### Cell viability and proliferation

DU-145 cells were cultured in 96-well plates (2 × 10^4^ cells/well) for 24 h before the serum-free medium was used and cells were treated with 100, 200, 500, 1000 μg/mL of fucoidan for another 24 h. Cell viability and proliferation were measured by 3-(4,5-dimethylthiazol-2-yl)-2,5-diphenyltetrazolium bromide (MTT, Amresco, Solon, OH) and 5-bromo-20-deoxyuridine (BrdU, Roche Diagnostics, Mannheim, Germany) incorporation assays, respectively, according to the manufacturer’s instructions.

### Cell migration

DU-145 cells were seeded into the insert of Transwell (Corning, Tewksbury, MA) at a density of 1 × 10^5^ cells/well, then cultured in serum-free culture media. Fucoidan (500 μg/mL) or vehicle (PBS) was added to the lower reservoirs. Cells were subsequently allowed to migrate across a collagen I-coated polycarbonate filter for 12 h at 37 °C. Non-migrated cells were removed from the top side of the filter by scraping. Migrated cells on the bottom side of the filter were subsequently fixed with 4% paraformaldehyde for 30 min and stained by hematoxylin solution (Beyotime, Shanghai, China) for 5 min. Cells in five random fields of each migration well were counted to determine the average number of migrated cells.

### Tube formation

24-well plates were coated with 300 μL Matrigel (BD, San Jose, CA) and incubated at 37 °C for 20 min to allow the Matrigel to solidify. DU-145 cells were plated at a density of 1 × 10^5^ cells/well and incubated with fucoidan (500 μg/mL) or vehicle (PBS) at 37 °C for 6 h. The cells were then photographed using a Zeiss digital camera. Tube formation was quantified by measuring the length of capillary structures using the software ImageJ (NIH, Bethesda, ML). Five randomly selected fields of view were photographed per well. The average value of the five fields was taken as the value for each sample.

### Animals and xenograft model

Athymic nude mice (5-week-old) were obtained from Charles River Laboratories (Beijing, China). Animals were housed in a temperature-controlled room (22 °C) with 12 h light/dark cycling under pathogen-free conditions, and had free access to food and water. The experimental procedures were approved by Institutional Animal Care and Use Committee of Ningbo No.2 Hospital. All animals were randomly divided into two groups (*n* = 6), and treated with vehicle (saline) or fucoidan (20 mg/kg) by oral gavage for 28 days. Subconfluent DU-145 cells were harvested by trypsin/EDTA treatment and washed with cold PBS by centrifugation, then resuspended in PBS and kept on ice before used. Tumor cells (1 × 10^7^ cells in 0.2 mL PBS) were injected subcutaneously into the mice. Tumor size was measured every four days by caliper, and tumor volume was calculated by the formula: 0.5 × (larger diameter) × (smaller diameter)^2^. At the end of experiment, the animals were sacrificed by CO_2_ euthanasia and the tumor tissues were harvested and weighted, then stored in −80 °C for further analysis.

### Hemoglobin assay

Concentration of hemoglobin in tumor tissue was determined using a Hemoglobin Colorimetric Assay Kit (Sigma-Aldrich) according to the manufacturer’s instructions.

### Real-time PCR

Trizol reagent (Takara, Dalian, China) was used for isolating total RNA of tumor tissue. 50–100 mg of tissue was directly lysed by adding 1 mL of Trizol reagent and homogenized using a homogenizer. Then 0.2 mL of chloroform was added, and the homogenized sample was incubated for 15 min at room temperature. Subsequently, RNA was precipitated by mixing with isopropyl alcohol. Total RNA yield was quantified by UV spectrophotometry measured at 260 nm. Then mRNA was isolated from total RNA by using Oligo (dT), and reverse transcribed into first-strand complement DNA (cDNA) and amplified using a PrimeScript 1st Strand cDNA Synthesis Kit (Takara). A total volume of 25 μL reaction mixture contained 2 μL of cDNA, 12.5 μL of 2 × SYBR Green 1 Master Mix (Takara, Dalian, China), and 1 μL of each primer. The PCR condition was as follows: pre-incubation at 95 °C for 30 s, followed by 40 cycles of denaturation at 95 °C for 5 s, and annealing/extension at 60 °C for 30 s using iQ5 Real-Time PCR detection System (Bio-Rad, Hercules, CA). The primers used were as follows [20]:

- CD31:
  - 5′-TATCCAAGGTCAGCAGCATCGTGG-3′
  - 5′-GGGTTGTCTTTGAATACCGCAG-3′
- CD105:
  - 5′-CCTTTGGTGCCTTCCTGATTG-3′
  - 5′-TGTTTGGTTCCTGG-GACAAGTTC-3′
- 18S:
  - 5′-GATGGGCGGCGGAAAATAG-3′
  - 5′-GCGTGGATTCTGCATAATGGT-3′

### Western blot

Tumor tissue was lysed with Protein Extraction Reagent (Beyotime), and protein concentration was determined by BCA reagent (Beyotime). About 20 μg of protein was separated in 10% SDS-polyacrylamide gel electrophoresis and transferred to a polyvinyl difluoride (PVDF, Millipore) membrane. After blocking with TBST containing 5% milk for 1 h, the membrane was incubated with antibodies against JAK, p-JAK, STAT3, p-STAT3 and GAPDH (Cell Signaling, Danvers, MA) overnight at 4 °C. After incubation in horseradish peroxidase-conjugated secondary antibody for 1 h, the membrane was exposed to Immobilon solution (Millipore) for band detection.

### Chromatin immunoprecipitation (ChIP)

An Agarose ChIP Kit (Pierce, Rockford, IL) was used to prepare nuclear extracts from tumor tissue homogenate and perform ChIP according to the manufacturer’s instructions. A ChIP-grade primary antibody against STAT3 was purchased from Cell Signaling. Immunoprecipitated DNA was purified with DNA Clean-Up Column (Qiagen, Hilden, Germany) and then quantitated by real-time PCR using PrimeScript RT-PCR Kit (TAKARA). The primers used were as follows [21]:

- *VEGF*:
  - 5′-CTGGCCTGCAGACATCAAAGTGAG-3′
  - 5′-CTTCCCGTTCTCAGCTCCACAAAC-3′
- *Cyclin D1*:
  - 5′-GTTGACTTCCAGGCACGGTT-3′
  - 5′-GATCCTCCAATAGCAGCAAACAAT-3′
- *Bcl-xL*:
  - 5′-CTGGGTTCCCTTTCCTTCCA-3′
  - 5′-TCCCAAGCAGCCTGAATCC-3′

### Statistical analysis

Data were analyzed and graphed by Prism 6.0 (GraphPad Software, La Jolla, CA), and presented as Mean ± standard deviation (SD). Significance of difference between groups was analyzed by performing two-way RM analysis of variance (ANOVA) for time course study, or one-way ANOVA with Dunnett’s multiple comparison test or unpaired Student’s *t* test for other studies. *P* value no more than 0.05 was considered statistically significant.

## Results

### Fucoidan inhibited viability, proliferation, migration and tube formation of DU-145 cells

**Fig. 1:**
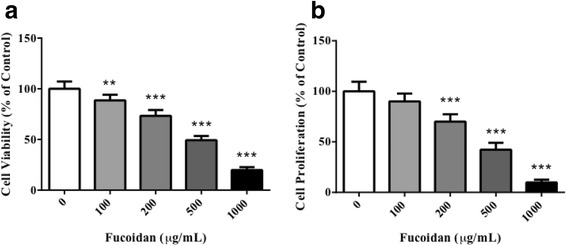
Fucoidan inhibited viability and proliferation of prostate cancer cells. DU-145 cells were treated with 100, 200, 500, 1000 μg/mL of fucoidan for 24 h. Cell viability (**a**) and proliferation (**b**) were measured by MTT and BrdU incorporation assay, respectively. ^**^ *P* < 0.01 vs. control, ^***^ *P* < 0.001 vs. control. All experiments were repeated at least three times

**Fig. 2:**
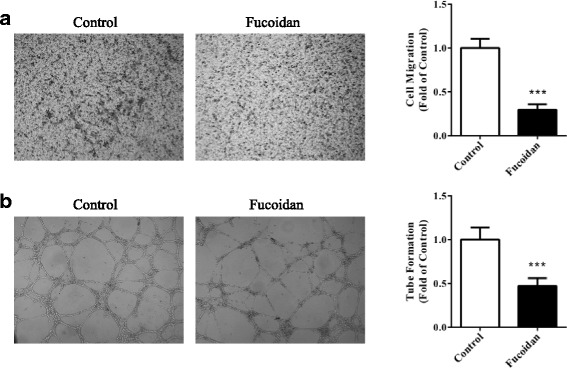
Fucoidan inhibited migration and tube formation of prostate cancer cells. **a** DU-145 cells were seeded into Transwell and treated with fucoidan (500 μg/mL) for 12 h. **b** DU-145 cells were seeded into Matrigel and treated with fucoidan (500 μg/mL) for 6 h. ^***^ *P* < 0.001 vs. control. All experiments were repeated at least three times

We treated DU-145 cells with different concentrations of fucoidan to assess its possible anti-angiogenic effects in vitro. 100, 200, 500 and 1000 μg/mL of fucoidan could inhibit viability of DU-145 cells in dose-dependent manner, with inhibition rate of 11.5%, 26.7%, 50.7% and 80.2% (Fig. 1a, *P* < 0.01, *P* < 0.001, *P* < 0.001, *P* < 0.001 vs. control, respectively). The IC50 dose of fucoidan was 497 μg/mL. Likewise, 200, 500 and 1000 μg/mL of fucoidan also inhibited proliferation of DU-145 cells in dose-dependent manner, with inhibition rate of 30%, 57.8% and 90.2% (Fig. 1b, *P* < 0.001 vs. control). Cell migration and tube formation are two critical steps in angiogenesis, therefore, we tested the efficacy of fucoidan in these in vitro assays. In Transwell assay, 500 μg/mL of fucoidan significantly inhibited the migration of cells to the other side (Fig. 2a, *P* < 0.001 vs. control); and in Matrigel assay, the dosage of fucoidan significantly reduced the length of formed tubes (Fig. 2b, *P* < 0.001 vs. control). All data showed that fucoidan inhibited in vitro angiogenesis.

### Fucoidan inhibited tumor growth and angiogenesis of prostate cancer xenograft

**Fig. 3:**
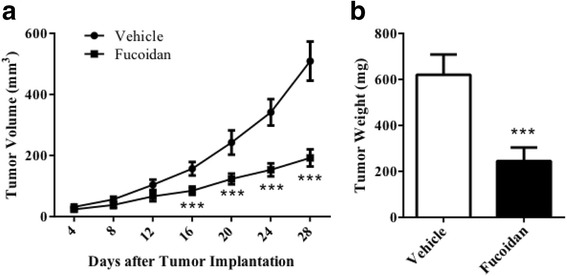
Fucoidan inhibited tumor growth of prostate cancer xenograft. Athymic nude mice were injected subcutaneously with DU-145 cells (1 × 10^7^ cells in 0.2 mL PBS), and treated with vehicle (saline) or fucoidan (20 mg/kg) by oral gavage for 28 days. **a** Tumor volume. **b** Tumor weight. ^***^ *P* < 0.001 vs. vehicle group. *N* = 6 for each group

**Fig. 4:**
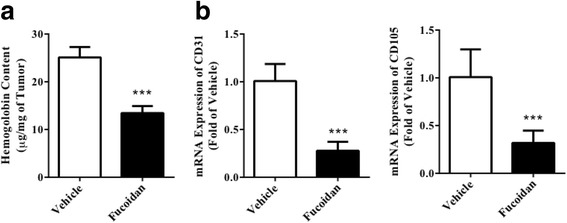
Fucoidan inhibited angiogenesis in tumor tissue. Tumor tissue from prostate cancer xenograft was isolated and homogenized for angiogenesis analysis. **a** Hemoglobin content determined by colorimetric method. **b** mRNA expression of CD31 and CD105 determined by real-time PCR. ^***^ *P* < 0.001 vs. vehicle group. *N* = 6 for each group

DU-145 cells were injected subcutaneously into athymic nude mice to induce ectopic xenograft model. 20 mg/kg of fucoidan could significantly hinder the tumor growth from day 16 post-tumor implantation (Fig. 3a). At the termination, tumor size in fucoidan group was 192.3 ± 28.1 mm^3^, while that in vehicle group was 509.2 ± 64.0 mm^3^ (*P* < 0.001). Likewise, tumor weight in fucoidan group was also significantly lower than that in vehicle group (Fig. 3b, 244.7 ± 58.8 mg vs. 620.0 ± 88.1 mg, *P* < 0.001). Then, we analyzed the vascular density in xenograft by hemoglobin assay and found that fucoidan significantly decreased hemoglobin content from 25.1 ± 2.2 μg/mg to 13.4 ± 1.5 μg/mg (Fig. 4a, *P* < 0.001). At the meanwhile, we determined mRNA expression level of CD31 and CD105, biomarkers of endothelium, in tumor tissue to find that both of them were also declined after fucoidan treatment (Fig. 4b, *P* < 0.001). All data showed that fucoidan hindered tumor growth by inhibiting angiogenesis.

### Effect of fucoidan on JAK-STAT3 pathway in tumor tissue

**Fig. 5:**
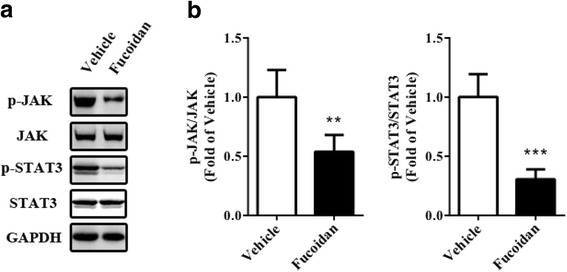
Fucoidan reduced phosphorylation of JAK and STAT3. Tumor tissue from prostate cancer xenograft was isolated and homogenized for Western blot. **a** Representative blot. **b** Statistical analysis of (**a**). GAPDH was used as a loading control. **P < 0.01 vs. vehicle group, ***P < 0.001 vs. vehicle group. *N* = 6 for eachgroup

**Fig. 6:**
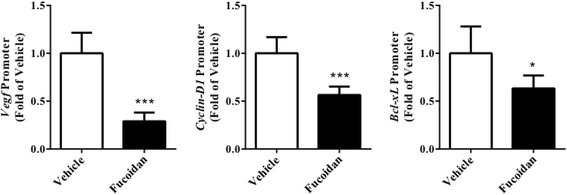
Fucoidan inhibited activation of STAT3-regulated gene promoters. The nuclear extract in tumor tissue was isolated and used to perform chromatin immunoprecipitation with an STAT3 antibody. The change of downstream promoters was analyzed by real-time PCR. ^*^ *P* < 0.05 vs. vehicle group, ^***^ *P* < 0.001 vs. vehicle group. *N* = 6 for each group

Considering JAK-STAT3 pathway is a target of angiogenesis-mediated cancer therapy, we continued to investigate whether the pathway was inhibited by fucoidan treatment. First, we analyzed the protein expression in tumor tissue by Western blot and find that phosphorylated JAK and STAT3 were both reduced after treatment (Fig. 5, *P* < 0.01 for JAK, *P* < 0.001 for STAT3). Next, we performed ChIP to investigate changes of STAT3-regulated gene promoters in the xenograft. The activation of *VEGF*, *Cyclin D1*, *Bcl-xL* promoters was significantly reduced after treatment (Fig. 6, *P* < 0.001 for *VEGF*, *P* < 0.001 for *Cyclin D1*, *P* < 0.05 for *Bcl-xL*).

## Discussion

In the past decades, many therapies, such as androgen-ablation therapy, prostatectomy, radiation therapy and cytotoxic chemotherapy, were developed to treat prostate cancer, but the subsequent adverse effects are also obvious [22, 23]. As a food-grade ingredient, fucoidan is extracted from marine plant. Previous clinical studies showed that long-term intake of fucoidan was safe in both healthy people and cancer patients [17, 18, 24]. In our study, we proved anti-tumor activity of fucoidan in both cell-based assays and mouse xenograft model, shedding new light for complementary and alternative therapy of prostate cancer.

Targeting angiogenesis is a new direction of cancer therapy [25]. Angiogenesis involves several sequential phases, in which sprout formation is initiated with the release of proteolytic enzymes from endothelial cells to degrade surrounding basement membrane, followed by cell proliferation and migration, finally the migrating cells form tube-like structures [26, 27]. In previous study, fucoidan was reported to Inhibit migration and invasion of A549 human lung cancer cell and tube formation of Hela cells in vitro [28, 29]. Furthermore, fucoidan reduced microvessel density and expression of VEGF in mice xenograft of 4 T1 mammary carcinoma cells [30]. Although Boo reported inhibitory effect of fucoidan on viability of PC-3 human prostate cancer cells, whether anti-angiogenic mechanism was involved was still unknown [19]. Here, we first reported inhibitory effects of fucoidan on proliferation, migration and tube formation of DU-145 prostate cancer cells, more importantly, we disclosed anti-angiogenic effects of fucoidan using a mouse xenograft model, in which hemoglobin assay and CD31 analysis directly proved fucoidan reduced vascular density in the tumor.

STAT3 is a candidate molecular target in angiogenesis-mediated therapy [31]. VEGF expression correlates positively with STAT3 activity in diverse human cancer cell lines [10]. An activated STAT3 mutant could up-regulate VEGF expression and stimulates tumor angiogenesis [10]. On the contrary, targeting STAT3 could block expression of VEGF induced by multiple oncogenic growth signaling pathways, and then inhibit tumor angiogenesis [32]. In this study, we also found reduction of STAT3 phosphorylation in tumor tissue, in which angiogenesis was inhibited by fucoidan.

As a transcription factor, STAT3 is phosphorylated to form dimers and then translocate to nucleus, where the dimers directly regulate the expression of genes responsible for survival (*Bcl-xL*, *Survivin*, *p53*), proliferation (*Myc*, *Cyclin D1/2*) and angiogenesis (*VEGF*, *HIF*) [31]. In this study, using ChIP, we disclosed reduced activation of *VEGF*, *Cyclin D1*, *Bcl-xL* promoters after fucoidan treatment, suggesting expression inhibition of these genes. VEGF is a vital regulator in angiogenesis and it is mainly secreted by tumor cells and targets VEGF receptor on endothelial cells to promote angiogenesis [33]. VEGF-mediated autocrine loop in endothelial cells is also an essential component of solid tumor angiogenesis [34]. Cyclin D1 is a protein required for progression through the G1 phase of the cell cycle [35]. Overexpression of cyclin D1 contributes to malignant properties of tumor cells by increasing VEGF production and decreasing Fas expression [36]. Bcl-xL, one member of Bcl-2 family, acts as an anti-apoptotic protein by preventing the release of mitochondrial cytochrome c to cytoplasm, which leads to caspase activation and programmed cell death [37]. Therefore, expression inhibition of *VEGF*, *Cyclin D1* and *Bcl-xL* could prevent angiogenesis and promote apoptosis to hinder tumor growth.

## Conclusions

Taken together, we first disclosed anti-tumor and anti-angiogenic effects of fucoidan, a food-grade ingredient, on prostate cancer in both cell-based assays and mouse xenograft model, as well as clarified a role of JAK-STAT3 pathway in the protection. All these findings provided novel complementary and alternative strategies to treat prostate cancer.

## Abbreviations

ChIP: Chromatin Immunoprecipitation
JAK: Janus kinases
STAT3: Signal transducers and activators of transcription 3
